# Dynamical Phenotyping: Using Temporal Analysis of Clinically Collected Physiologic Data to Stratify Populations

**DOI:** 10.1371/journal.pone.0096443

**Published:** 2014-06-16

**Authors:** D. J. Albers, Noémie Elhadad, E. Tabak, A. Perotte, George Hripcsak

**Affiliations:** 1 Department of Biomedical Informatics, Columbia University, New York, New York, United States of America; 2 Department of Mathematics, Courant Institute of the Mathematical Sciences, New York, New York, United States of America; Universitat Pompeu Fabra, Spain

## Abstract

Using glucose time series data from a well measured population drawn from an electronic health record (EHR) repository, the variation in predictability of glucose values quantified by the time-delayed mutual information (TDMI) was explained using a mechanistic endocrine model and manual and automated review of written patient records. The results suggest that predictability of glucose varies with health state where the relationship (e.g., linear or inverse) depends on the source of the acuity. It was found that on a fine scale in parameter variation, the less insulin required to process glucose, a condition that correlates with good health, the more predictable glucose values were. Nevertheless, the most powerful effect on predictability in the EHR subpopulation was the presence or absence of variation in health state, specifically, in- *and* out-of-control glucose versus in-control glucose. Both of these results are clinically and scientifically relevant because the *magnitude* of glucose is the most commonly used indicator of health as opposed to glucose dynamics, thus providing for a connection between a mechanistic endocrine model and direct insight to human health via clinically collected data.

## Introduction

Intuitively we know that many macroscopic human traits, or phenotypes, including many diseases, are a composite of many interacting variables and systems spanning scales from the molecular to the social. Moreover, we also understand and observe that human phenotypes are time-dependent, or dynamic; diseases evolve in time, the probability of acquiring diseases, including those with a strong genetic component, can change in time, and general physical characteristics change with age. Yet, for the most part, the dynamic nature of phenotyping has been neglected. We hypothesize that the current lack of phenotypes that are dependent on temporal characteristics of humans is due in part to the fact that important temporal features that affect phenotypic differences require data sets that span large populations and diverse time scales such that differences can be observed. Collecting such data solely for the sake of science is likely too expensive and intrusive to ever be done on a large scale. The solution to this problem is to use data that are automatically collected for a different purpose, electronic health record (EHR) data. Nevertheless, using EHR data in a more basic science context requires a better integration between physiology and clinical practice to both drive useful innovation and to cope with health-care-process dependent data complexities [1].

*Human physiology* focuses on the mechanical, physical, and biochemical functioning of humans. Physiology uses basic science machinery (e.g., molecular biology, mathematics) and well understood phenotypic definitions in a very narrow, precise, and controlled way. For example, the data that human physiologists collect and study are captured in highly controlled environments from very carefully chosen and controlled individuals usually over relatively short time periods. Discovering and quantifying diverse phenotypes and their evolution in time is difficult while remaining within the context of human physiology because such controlled and small populations of data over limited time scales do not contain the potential for resolving diverse and evolving phenotypes.

*Clinical practice* involves the practical management of patients in a hospital or other care center. Clinical phenotypes are often complex, broadly and descriptively defined, and their definition is driven and guided to help identify and treat a macroscopic observable condition such as a disease. While physiology is applied in some clinical practice environments, physiology is often used for intuition rather than for concrete decision making. The focus of clinical research is primarily practical because the clinicians are required to help the patients with a degree of immediacy that makes it difficult perform some types of research. Discovering and precisely quantifying diverse phenotypes and their evolution in time is difficult solely within the context of clinical practice because of the immediate need for clinical treatment of individuals.

Despite the fact that physiology and clinical practice are highly related, currently they are not well integrated. More bluntly, clinical practice and physiology are not unified in the same way that engineering and physics are, despite the fact that physiology forms the scientific basis for many medical practices and treatments in the same way that physics is used to construct bridges. At its heart, the difference lies in the lack of computation that might integrate or translate complex physiologic information into clinically actionable knowledge. Engineers use physics to precisely *calculate* features required to construct bridges whereas doctors rarely use physiology to precisely compute features required to give care to individuals. One of the aims of personalized medicine is to customize treatment for an individual based on individual situations and characteristics. Integrating the mechanistic knowledge of how physiology affects health state into clinical practice in a tangible way, allowing for differences in people to be accounted for and used to predict future health, will make personalized medicine possible.

We feel that the time has come to begin integrating physiology with clinical practice in a more explicit way. Moreover, we assert that the integration should occur via a common data set, EHR data that are collected for clinical purposes. EHR data are comprised of all the information clinicians collect, are complex in nature (e.g., lab values, billing information, and narrative text), span many scales in space and time, are not collected in a controlled environment and therefore contain many complex biases [2] [1], and are large in size. In “Toward Precision Medicine: Building a Knowledge Network for Biomedical Research and a New Taxonomy of Disease” [3] (cf. Fig. S-1, page 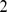) the authors call for the creation of a new taxonomy (e.g., data driven phenotypes) as a way of pushing both clinical practice and basic biological understanding of humans forward in a data driven manner:

Creation of a New Taxonomy first requires an Information Commons in which data on large populations of patients become broadly available for research use and a Knowledge Network that adds value to these data by highlighting their inter-connectedness and integrating them with evolving knowledge of fundamental biological processes. [3].

Because EHR data are collected in a clinical environment, they contain a broad population, and they can act as a practical bridge between basic science and clinical practice because the data that are available in an EHR represent what information can be, and are in practice, measured by clinicians. The hope is then that the use of this common data set will form a feedback loop where clinically collected data suggest physiologic problems to solve, which drives now physiologic understanding, which drives new clinical treatments and measurements, that again motivate new physiologic problems, etc.

### Dynamical phenotyping

**Figure 1:**
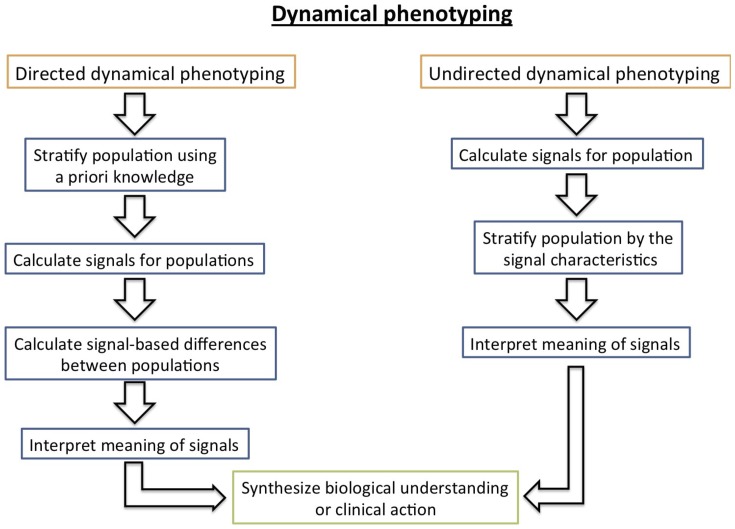
Depicted above are the two different dynamical phenotyping strategies, *directive dynamical phenotyping* where the population is stratified and then characterized by differences in dynamics, and *undirected dynamical phenotyping* where a complex population is stratified by differences in dynamics.

With a data set spanning a broad population over a long time period, there are many options available for stratifying the population into different categories that can be understood physiologically. Here we develop a method based on the dynamical differences of subpopulations, where dynamical difference is defined by inter-individual differences in signals generated use nonlinear time series analysis techniques. As shown in Fig. 1, there are two ways to conceive of *dynamically phenotyping* a population. *First*, one can employ *directed dynamical phenotyping* which begins by stratifying the population *a priori* (e.g., diabetics and non-diabetics), generating signals for the different populations that show difference, and then explaining the sources of those differences. *Second*, one can employ *undirected dynamical phenotyping* which begins with a complex population for which temporal signals are calculated and then used to stratify the population. The final step in both cases involves explaining the signal sources and the source of the signal differences. We believe that oscillating between these approaches will drive a hypothesis generation and hypothesis refinement feedback loop that will further both clinical and basic biological understanding.

Narrowing the scope, in this paper we are concerned with understanding variations in endocrine dynamics in humans over a time scale of days to weeks and how differences in glucose dynamics on this time scale can be explained using mechanistic glucose/insulin models [4] [5]. In Albers *et al* [6] we employed a directed dynamical phenotyping approach using two populations, glucose values from a population of 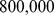 random patients, and glucose values of a population of 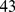 tube fed, comatose patients in an ICU setting. This paper demonstrated that while raw glucose values could not be used to separate the two populations based on their glucose dynamics, a derived value (the time-delayed mutual information (TDMI), cf. section 0.4) could. Specifically, the TDMI of the random and ICU populations differ in that the TDMI for the random population does display a diurnal cycle, and that diurnal cycle is driven by structured (conditionally random) eating habits. These results were explained and confirmed using a mechanistic glucose/insulin model [4], which in turn verified the mechanistic physiologic model well beyond the context within which it was originally designed to apply. Nevertheless, within the population of 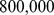 patients, there was diversity within the TDMI-based diurnal signal. Whereas, Albers *et al* [6] represents a directed dynamical phenotyping approach, this paper represents a substantial refinement using an undirected dynamical phenotyping approach aimed at understanding the nature of the diversity within a population of random humans not controlled within an ICU context. To explain the diversity of diurnal TDMI signal we must use both a mechanistic glucose/insulin model and the full breadth of the EHR data set via natural language processing (NLP) analysis and manual review of patient records.

## Materials and Methods

### 0.1 Ethics statement

This work was approved by the Columbia University Institutional Review Board. Informed consent was waived by the Institutional Review Board for this retrospective research.

### 0.2 Data assess statement

Unfortunately, the data for this study cannot be made publically available because the detail and complexity of the data put it at risk for re-identification. Similar data are publically available from the PHYSIONET and MIMIC data repositories.

### 0.3 Methodological background

Adopting the undirected dynamical phenotyping approach requires, as a starting point, dynamical signals that register diversity within a population. Here we rely on two results from Albers and Hripcsak [7] that provide the necessary distribution of signals that provide the necessary diversity. *The first result* says that the TDMI for a population can be computed via two different ways of collecting the same measurements, and that the outcome of those calculations are identical (up to bias) if and only if the population is homogeneous (cf. conjecture 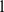 of Albers and Hripcsak [7]). *The second result*, shown in Fig. 6*a* of Albers and Hripcsak [7] shows that, for the EHR subpopulation used to demonstrate the workings of the TDMI calculation in the EHR-data context, the given subpopulation was *heterogeneous when observed over short time periods* and *homogeneous over longer time periods*. That this is the case means that there is *meaningful variation* in the distribution of TDMI values for patients in this population for time scales of 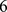
hrs and less. This in turn implies that we can stratify this patient population by TDMI calculated over for time separations of 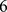
hrs. The primary foci of this paper are to: *(i)* begin to investigate whether variation in predictability for a population, quantified by the TDMI, can be used to stratify the population, and *(ii)* begin to understand the meaning of the strata of populations cleaved by predictability.

To stratify the EHR subpopulation and then understand the strata, we will use four tools: **(i)** clustering of the TDMI distribution; **(ii)**, the TDMI variation of a mechanistic model of glucose/insulin dynamics [4] [5] under parameter variation; **(iii)** an NLP analysis of the patient notes; and **(iv)**, a manual review of the patient records. More constructively, we begin with the distribution of TDMI values for a population of patients knowing from previous work that the first order dependence of this distribution is nutrition [6].

To separate the population based on the variation within the TDMI distribution we cluster the TDMI distribution using flow-based clustering (FBC) [8]. We then use the glucose/insulin model to explain the variation observed in the TDMI from a mechanistic modeling perspective. Finally, we use both NLP analysis and manual review to interpret the meaning of the clusters based on the patient notes and records to verify the results of the comparison with the mechanistic model and to endow the clusters with a clinical and physiologic interpretation.

### 0.4 Time-delay mutual information

The TDMI [9], [10] in its most simple form is given by:
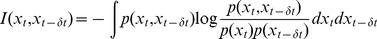
(1)where 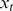 and 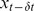 represent the same variable measured at 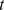 and 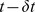 respectively; these collected pairs of variable form ensembles, and 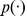 denotes the probability density function (PDF) defined by those ensembles. Note that the TDMI captures linear and nonlinear correlations in time, which differs from, say, auto or linear correlation calculations. Under most circumstances, the TDMI is calculated for an individual. For reasons that will become clear shortly, we want to calculate the TDMI for a population, and for the individuals within the population. There are roughly two different explicit means of calculating the TDMI for a population. First, one can calculate an *average TDMI*, which is just an average of the TDMI calculated for individuals; in this case 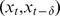 would represent all the pairs of measurements separated by 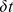 *for an individual*. This calculation yields both a distribution of TDMI values for the population, and a population average. Second, one can calculate the TDMI for the aggregated population; in this case 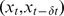 represents a collection of *all the intra-patient pairs of points in the population of time series separated by a time* 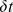 aggregated together. The average and aggregate TDMI for a population are equal if and only if the populations are identical in distribution [7]. It is known from previous work (cf., Fig. 6*a* of Albers and Hripcsak [7]) that the population we use in this paper is *heterogeneous* for 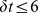
hrs and *homogeneous* for 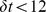
hrs, thus implying that on relatively fast time-scales, the population appears diverse and can be stratified.

More practically, the TDMI is a unit-less quantity; a TDMI of 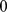 (within bias) implies that there is no correlation between sequential values in a time series for a given 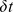. TDMI values begin to become important when they exceed the expected bias associated with calculating the mutual information, which is approximately 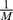 where 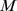 is the number of pairs of points used to estimate the TDMI (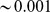 in this experiment). With a perfect correlation between sequential values, the TDMI will be equal to the entropy (or auto-information) of the series, which is numerically equal to the TDMI at 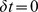 (and is calculated automatically as part of the experiment). In this experiment the entropy was about 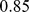 and represented the maximum TDMI. (In most of our experiments, the entropy is in the 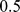 to 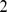 range.) Note that perfect correlation of a constant function (implying PDFs that are 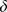 functions) yields a TDMI of zero for all 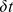. Finally, to calculate the TDMI, one must estimate the joint and marginal PDFs, here we used a kernel density estimation (KDE) routine [11] implemented on MATLAB.

### 0.5 The glucose/insulin model

We use the model developed by Sturis *et al* [4] which consists of six ordinary differential equations (ODEs), specifically:
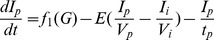
(2)

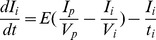
(3)

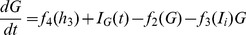
(4)and a three stage linear filter:
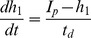
(5)

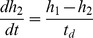
(6)

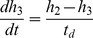
(7)where the state variables correspond to: 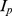, plasma insulin; 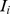, remote insulin; 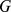, glucose; and 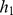, 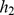 and 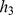 which corresponds to three parameterized delay processes. The *major* parameters include: *(i)* 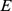, a rate constant for exchange of insulin between the plasma and remote compartments; *(ii)* 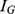, the exogenous (externally driven) glucose delivery rate; *(iii)* 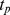, the time constant for plasma insulin degradation; *(iv)* 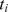, the time constant for the remote insulin degradation; *(v)* 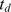, the delay time between plasma insulin and glucose production; *(vi)* 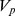, the volume of insulin distribution in the plasma; *(vii)* 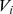, the volume of the remote insulin compartment; *(viii)* 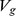, the volume of the glucose space; *(ix)* 
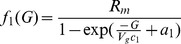
, insulin secretion; *(x)* 
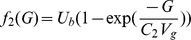
, insulin-independent glucose utilization; *(xi)* 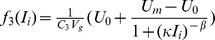, insulin-dependent glucose utilization (
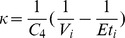
); and *(xii)* 
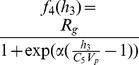
, insulin-dependent glucose utilization.

One of the major advancements in the above model over more minimal models is how glucose, 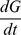 is modeled. Glucose is added both by exogenous nutrition that is independent of insulin (e.g., via eating), 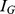, and by insulin dependent processes controlled by the liver via glycogenolysis and gluconeogenesis (e.g., via exercise [12]). The rate at which glucose is added to the blood by liver-related processes is controlled 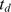, the delay time between plasma insulin and glucose production. Similarly, glucose is removed by insulin independent glucose utilization, 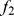, and insulin dependent glucose utilization, 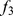.

The meaning and nominal values of all these variables, except 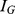 which is discussed below, and constants are summarized in table 1. Note that in Sturis *et al* [4] they did perform a sensitivity analysis, meaning that they numerically demonstrated that the *dynamic types* (e.g., periodic orbit, fixed point, etc.) were stable under small parameter perturbations.

**Table 1:** Full list of parameters for the glucose/insulin model [4] used in this paper; note that these are the model parameters we us in this paper.

Glucose model parameters and their TDMI relationships					
Parameter	nominal value	meaning	linear correlation, p-value	linear regression (slope)	effect on TDMI
*V_p_*	3 l	plasma volume	0.44, 0.05	4.6×10^−5^±6×10^−4^	—
*V_i_*	11 l	insulin volume	0.28, 0.21	2.4×10^−4^±10^−5^	—
*V_g_*	10 l	glucose space	0.9, 10^−8^	4×10^−4^±10^−4^	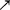
*E*	0.2 l min^−1^	exchange rate for insulin between remote and plasma compartments	0.15, 0.5	1.3×10^−5^±6×10^−6^	—
*t_p_*	6 min	time constant for plasma insulin degradation (via kidney and liver filtering)	−0.67, 10^−3^	−1×10^−4^±8×10^−4^	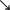
*t_i_*	100 min	time constant for remote insulin degradation	0.13, 0.57	1×10^−5^±6×10^−4^	—
*t_d_*	12 min	delay between plasma insulin and glucose production	−0.82, 10^−4^	−5×10^−4^±2×10^−3^	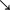
*R_m_*	209 mU min^−1^	linear constant affecting insulin secretion	0.72, 10^−4^	−1×10^−4^±7×10^−4^	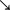
*a*_1_	6.67	exponential constant affecting insulin secretion	0.88, 10^−7^	7.4×10^−4^±3×10^−3^	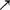
*C*_1_	300 mg l^−1^	exponential constant affecting insulin secretion	0.87, 10^−7^	9×10^−4^±3×10^−10^	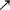
*C*_2_	144 mg l^−1^	exponential constant affecting IIGU	−0.04, 0.86	4×10^−6^±6×10^−4^	—
*C*_3_	100 mg l^−1^	linear constant affecting IDGU	0.8, 10^−6^	−5×10^−4^±2×10^−3^	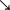
*C*_4_	80 mU l^−1^	factor affecting IDGU	0.16, 0.47	2×10^−4^±7×10^−4^	—
*C*_5_	26 mU l^−1^	exponential constant affecting IDGU	0.76, 10^−5^	1×10^−4^±7×10^−4^	—
*U_b_*	72 mg min^−1^	linear constant affecting IIGU	0.87, 10^−7^	6×10^−4^±2×10^−3^	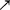
*U*_0_	4 mg min^−1^	linear constant affecting IDGU	0.85, 10^−7^	3×10^−4^±1×10^−3^	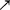
*U_m_*	94 mg min^−1^	linear constant affecting IDGU	0.028, 0.9	2×10^−6^±6×10^−4^	—
*R_g_*	180 mg min^−1^	linear constant affecting IDGU	−0.86, 10^−7^	−2×10^−3^±9×10^−3^	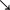
*α*	7.5	exponential constant affecting IDGU	−0.84, 10^−6^	−6×10^−4^±3×10^−3^	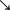
*β*	1.77	*exponent* affecting IDGU	−0.25, 0.26	−2.4×10^−4^±6×10^−4^	—

The delivery of nutrition or the *exogenous glucose delivery rate*, 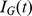, is an external driving and is the most dominant force in glucose/insulin dynamics. Previously we have considered five different feeding patterns [6]. Of those five different feeding patterns, here we will use the *noisy-periodic individual* because this nutrition pattern most accurately represents a human population eating regular, but not exactly periodic, meals.

To construct mealtime feeding, define the mealtime set 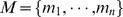, where the 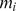
's represent times over a 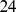-hour interval, and 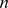 is the number of meal times within a 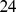-hour period. The exogenous glucose delivery rate at the current time, 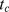, is defined by a function:
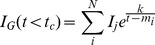
(8)where 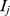 is the peak rate of delivery of glucose for a given individual 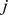 at time 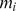, 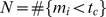 represents the total number of meals that have passed by time 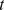, and 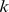 is the decay constant (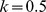). The decay constant determines that the meal is digested over two hours, a time that is considered realistic [4]. Next, fixing 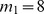, 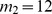, and 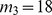, define the mealtimes of the *noisy individual*, 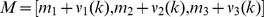, where 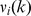 is a uniform random variable on the interval 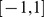 and 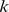 represents an integer day (implying that 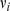 changes every day). Formally the *noisy-periodic individual*, 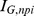), is defined by:
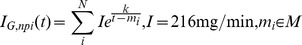
(9)


*The first statistical order*, the TDMI mean signal per 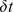 bin, was accurately reproduced for a broad EHR population [6] by the model we use here. Moreover the model predicted the difference between continuously (enterally/tube) fed patients and patients who acquired nutrition more normally [6], implying both that the model represents humans reasonably well and that nutrition is the most important factor driving the TDMI signal. Here we are really working to understand the higher order statistical factors that affect the predictability of glucose as quantified by the TDMI — meaning, we are trying to understand the sources of variance in the TDMI as they relate to human dynamics and health.

The ODEs were integrated over time-periods ranging from nine days to three weeks. A standard fourth-order Runge-Kutta integration routine, with a step-size of 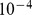, was used.

### 0.6 Flow-based clustering

Clustering a raw time series is relatively uncommon and can be complex because nonstationary and measurement properties (frequency, number of measurements, non-uniformity of measurements, etc.) can affect the ability to resolve modeled states and can affect the stability of parameter fittings of time series. If system parameters change in time, enough data must be collected and the model must be fit over time scales short enough such that the system is essentially stationary. Moreover, many models can be unstable relative to small changes in parameters or data; when using real data that is constrained by the ability to measure, the instability in models can be exacerbated. For example, a time series fit to a polynomial function is often unstable — small perturbations in parameters can wildly change the qualitative observed dynamics [13]. Therefore, in the time-series context what is more often done, and what we do here, is to derive a value from the time series that is stable (e.g., TDMI) and then cluster that value explicitly [14] [15]. We have applied a similar methodology in the context of cross-correlation coefficients using hierarchical clustering [1]; not surprisingly in that work we found that the clusters could be dependent on clustering method and non-hierarchical methods such as k-means did not yield interpretable results.

Given a starting point of stationary collections of features, the clustering problem consists of partitioning a set of observations into 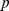 clusters 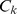 with common traits [16]. The most general way to characterize these traits is through a probability density 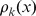, which specifies how likely it is to find a sample with observables 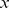 in the class 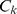.

Given one such probability density for each class, the posterior probability 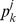 that the observation 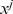 belongs to the class 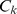 follows from Bayes formula,
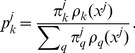
(10)


Our procedure [8], flow-based clustering (FBC), is based on fluid-like flows in feature space that cluster a set of observations by transforming them into likely samples from 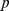 isotropic Gaussians, where 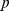 is the number of classes sought. The map 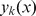 that transforms the density 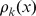 of class 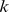 into a Gaussian 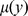 automatically provides an estimation of the underlying density, through the change of variables formula
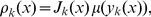
(11)where 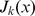 is the Jacobian of the map.

The parameter fitting is carried out using an expectation-maximization (EM) approach. Throughout the algorithm, each observation is softly assigned to each class, through the posterior that it belongs to it under the current density estimation for the various classes — this is the E step. The observations act as Lagrangian markers, or free floating buoys, that move with the flows at different rates depending on the current strength of the assignment to the corresponding class that determines the strength and direction of the flow — this is the M step. This procedure allows us to integrate the expectation-maximization methodology into a descent framework, based on the likelihood function
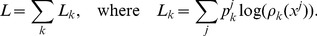
(12)


**Figure 2:**
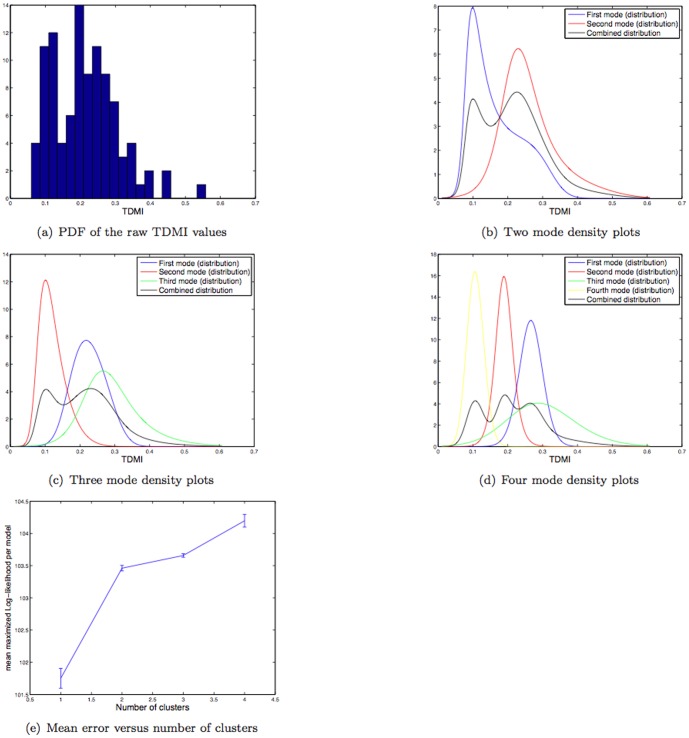
Depicted above are: (a) the histogram of the raw TDMI of glucose time series for 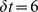 hrs for the population of 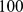 patients; (b) the 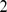 mode FBC model of the TDMI distribution; (c) the 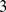 mode FBC model of the TDMI distribution; (d) the 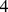 mode FBC model of the TDMI distribution; (e) variation in the distribution (as quantified by the mean and variance) of the log-likelihood for models with 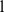
–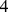 modes.

In the E step, the procedure starts by assigning each observation 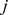 a nearly uniform prior 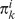, with a small random bias towards one class so as to break the symmetry among classes. Then, in the M step, a map depending on parameters 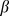 is proposed, and the parameters are chosen so as to maximize the likelihood 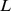. As the observations start to cluster into classes as the EM is iterated, their posteriors become sharper; it is these posteriors 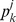 that weight each observation in the likelihood function and specify which data belong to which class. The plot in Fig. 2(e) depicts the final value of 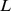 (the mean among all 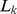) for various numbers of clusters.

### 0.7 Dynamical phenotyping using EHR data

In the introduction we proposed the concept of dynamical phenotyping (cf. Fig. 1) which we then split into directed and undirected types of dynamical phenotyping. In the context of EHR data, both of these approaches have pitfalls. Directed dynamical phenotyping is difficult even when equipped with an intuitive phenotype construction because EHR data contain complex biases [2], are collected in uncontrolled environments, have complex reasons for existence whose intent for measurement may carry unintended consequences [1], and do not uniformly represent all individuals. For example, one may want to contrast glucose dynamics in diabetics (types 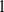 and 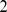) with healthy non-diabetics. However, there is a great deal of diversity in the health state and glucose management within the various diabetic populations; as we will see, enough to drown out a diabetic/non-diabetic signal. Moreover, uniformly healthy non-diabetics are rarely measured and thus do not have enough data to be compared with sicker patients who are measured more frequently. Finally, this approach builds in intuitive bias *a priori* which can limit results and potential for discovery. Similarly *discovering* a stratification of a complex population using undirected dynamical phenotyping can be difficult because of the potential diversity within the population; the lack of a narrowed population that can induce bias that can confound results simply because there can be too many categories to resolve for a given data set. To mitigate these pitfalls, we advocate for oscillating between both approaches to refine the populations and narrow the diversity of potential sources of signals while allowing for new, surprising information to be found. Previously, neither of these approaches have been concretely applied.

#### 0.7.1 Columbia University Medical Center data set composition

The data set we use here was not filtered or carefully selected in anyway other than the criteria that the patients are the 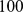 patients with the most glucose values in the Columbia University Medical Center (CUMC) EHR at the time of collection.

**Figure 3:**
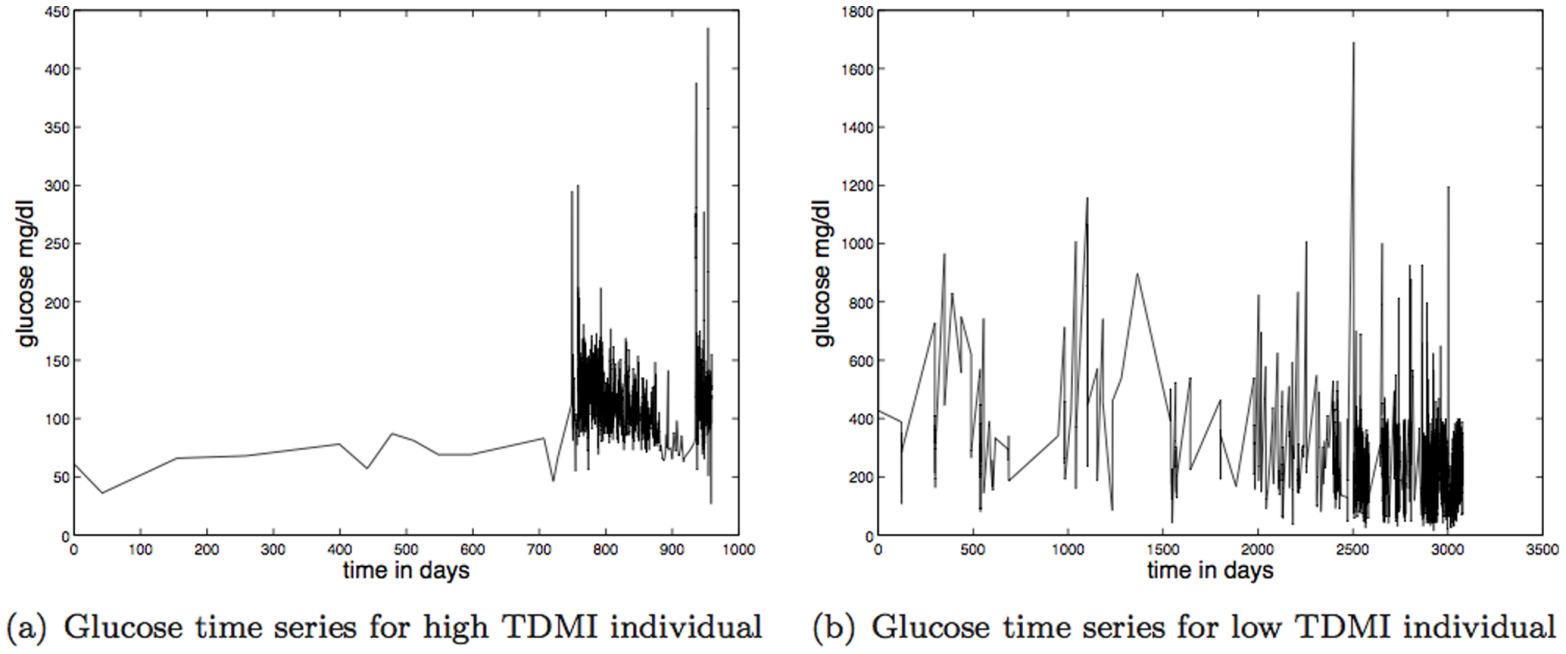
Depicted above are: (a) the glucose time series of an individual with high TDMI, 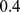, in the 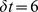
hrs bin — this individual falls into cluster two; (b) the glucose time series of an individual with low TDMI, 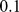, in the 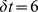
hrs bin — this individual falls into cluster one.

**Figure 4:**
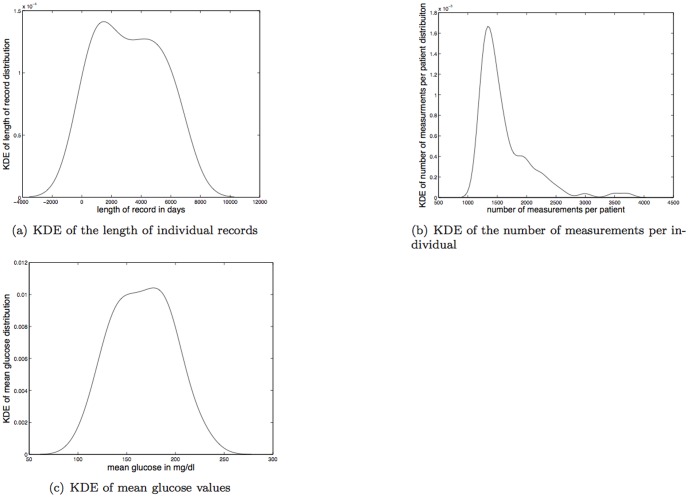
Depicted above are: (a) KDE of the length of individual records; (b) KDE of the number of measurements per individual; (c) KDE of the mean glucose per record.

There is considerable diversity within this data set; two example time series, one with high TDMI and one with low TDMI are shown in Figs. 3(a) and 3(b) respectively. The mean length of record is 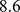 years with a maximum of 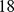 years and a minimum of 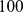 days; the kernel density estimate (KDE) of the lengths of records is shown in Fig. 4(a). The mean number of measurements per individual is 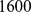 with a minimum of 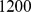 and a maximum of 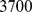; the KDE of the number of measurements can be found in 4(b). There is very little correspondence (e.g, linear correlation) between number of measurements and length of record or glucose value [17]; for example, the individual with the most measurements had one of the shortest records in time. The mean-mean glucose value for this data set was 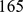 mg/dl with a maximum mean glucose of 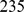 mg/dl and a minimum mean glucose of 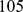 mg/dl; the KDE of mean glucose values per individual can be found in Fig. 4(c).

Because EHR data are not collected in a controlled environment, it is important to give a flavor of the complicated nature of the composition of EHR data as a data source. To demonstrate this, consider the hypothesis that diabetics would have the most frequently *recorded* glucose values. While a careful verification of this hypothesis is a substantial research question, we can make a rough inference into the validity of such a statement. Within this data set, 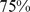 have at least one billing code for diabetes [7]. Thus, at least 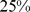 of these patients are *not* diabetic at all. One can imagine many plausible reasons for this. One example might be that a substantial portion of the glucose measurements come as part of a panel with seven other measurements, so many of the glucose measurements could be measured as part of a routine for caring for particularly sick patients, such as patients admitted for congestive heart failure. Thus, stratifying such data could be done in a nearly infinite number of ways. The point of this discussion is that EHR data are unpredictably complex and surprising and thus making any unverified assumptions about EHR data can lead to plausible, but false conclusions.

The diversity according to billing, or ICD9 codes can be found in Albers *et al* [7].

Finally, the patient records we study include patient notes that consist of both structured (e.g., forms) and unstructured text documenting events such as admissions, discharges, surgeries, radiology visits, etc. Of the 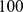 patients, 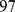 of the patients had notes (we do not know whether the remaining three patients had no notes, or whether these patients' notes were not accessible, which can happen for a variety of reasons). A full description of the composition of the notes will be discussed in the results section.

## Results

### 0.8 Clustering the population by the TDMI distribution for 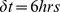


The distribution of TDMI values, shown in Fig. 2(a), is multi-modal, implying that that there is separable diversity within the population that is captured by the TDMI. The first step toward understanding the source of the multi-modality is to cluster the modes, which we will accomplish using FBC. Three examples (recall that FBC is dependent on the initial conditions of the clustering routine) of the resulting FBC of the TDMI distribution assuming two, three and four modes are shown in Figs. 2(b), 2(c), and 2(d) respectively. The respective variation in the distribution of the log-likelihood for the different models (e.g., the goodness of fit) with differing number of clusters is shown in Fig. 2(e). Based on these results one can observe that: **(i)** the variance is minimized with 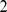 and 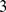 clusters, and is relatively high for 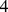 clusters; **(ii)** the log-likelihood is maximized for 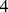 clusters; and **(iii)** there are big jumps in the goodness of fit between 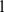 and 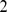 clusters, and 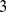 and 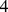 clusters and there is a relative plateau for 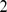 and 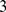 clusters. This analysis yields the conclusion that four clusters will yield the best separation, but the difference between 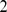 and 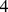 clusters is not substantial, especially given the fact that the log-likelihood must increase when the number of clusters is increased.

Visual inspection of the *plots* of the empirical and model distributions yields a different interpretation. Begin by noting how representative the final distribution of each fit is with respect to the original distribution; the original distribution seems to have two peaks and a single long tail, which is mimicked well by both the 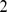 and 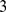 mode models. Furthermore, the third mode in the three mode model, and the fourth mode in the four mode model both model the tail of the distribution, yet allowing for substantial overlap in probability with the other classes. This implies that the two mode model may be the most useful for separating the populations. In all cases, there is substantial overlap in probability between distributions. Concentrating on the two mode model (Fig. 2(b)), note how much of probability of mode one overlaps with the support (including the location of the maximum probability) of the second mode. This implies that the middle of the TDMI distribution will likely be difficult to separate into different classes because there is too much probability mass overlap between the modes. This interpretation makes intuitive sense given that much of the mass of the TDMI distribution is near the overlap between modes and that there can be many reasons for a given TDMI value.

*It is important to remember* that we are, in essence, attempting to understand the mapping between a *one-dimensional* TDMI distribution or value and a physiologic explanation that may be of *much higher dimension*. In this context, over-fitting with too many clusters will likely yield poor results. Because of this, it is likely that the best stratification we can achieve with a single variable will be bi-modal and will correspond to individuals with high and low TDMI values. Therefore, in this work we will concentrate on understanding two phenotypes, patients with high and low TDMI values.

### 0.9 Interpreting the meaning of the dynamical clusters

#### 0.9.1 Static analysis of the 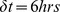 TDMI distribution

Before pushing on to the dynamical explanations for the clusters, it is important to rule out *static* explanations for the TDMI clusters. Potential explanations for the variation in the TDMI peak at 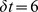
hrs could be due to correlations between a *static variables* such as the mean, standard deviation, or number of glucose measurements per patient. We find that there is no significant linear relationship between the TDMI and either the mean glucose (LC of 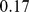, p-value for the hypothesis of no correlation against their being correlation, 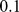) or the standard deviation of glucose values (LC of 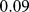, p-value for the hypothesis of no correlation against their being correlation, 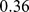). While there does appear to be a relationship between the TDMI and the number of measurements per patient (p-value for the hypothesis of no correlation against their being correlation, 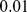, number of measurements ranged from approximately 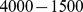 per patient), the relationship was extremely weak (LC of 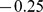). The overall point is that glucose value or variance is *not* a good proxy for predictability for this set of patients [17].

#### 0.9.2 Dynamical systems, mechanistic physio-model analysis of the 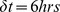 TDMI distribution

To deduce the physiologic mechanisms that can be the source of the broad multi-modality of the TDMI distribution shown in Fig. 2(a), we observe how the TDMI distribution, estimated using time-series generated by the mechanistic physiologic model introduced in section 0.5, varies when the parameters of the mechanistic physiologic model are varied. This analysis also yields predictions of fine scale structure in the TDMI distribution that cannot be resolved with the data we use in this paper, but that will hopefully be resolvable using a more refined and filtered data set in the future.

**Figure 5:**
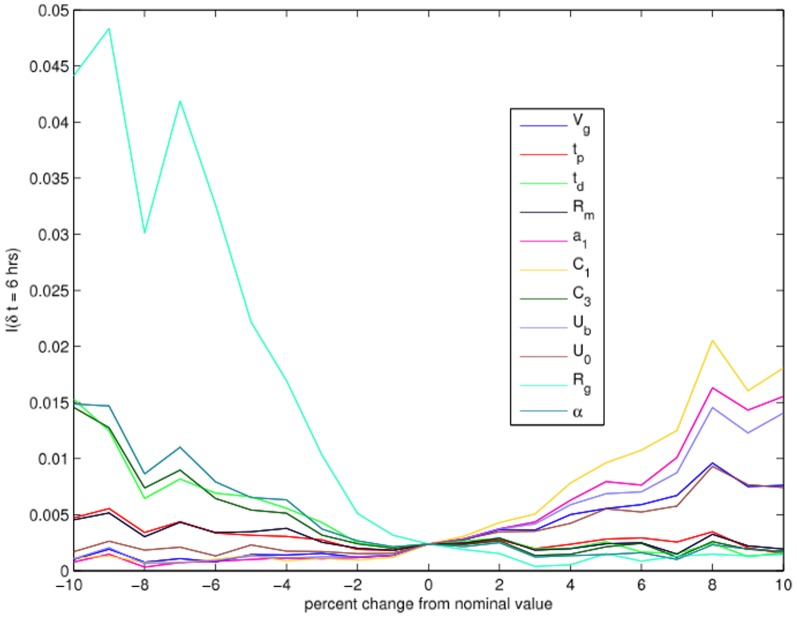
Parameter variation plot versus predictability (TDMI) for selected parameters.

**Variation of TDMI with variation of parameters:** To understand what the TDMI, which quantifies predictability, implies about physiology, we must understand which variables control the width of the TDMI distribution (i.e., the variance) for a given time separation. We investigate this by performing a parameter variation TDMI-based analysis where we vary each of the 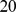 parameters of the model systematically within 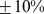 of their nominal values (with 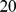 discrete increments), and then observe the changes in the TDMI. The effects of the variation of parameters have on the TDMI are then quantified in two steps. First, we calculated the linear (Pearson's) correlation coefficient (LCC) and its associated p-value to ascertain the strength of the linear relationship between the parameter variation and the TDMI. The closer the LCC is to 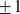, the tighter the distribution lies about the line of best fit whereas the closer the LCC is to zero, the weaker the linear relationship is (note that an LCC close to zero essentially implies no linear relationship, but nothing else). Further recall that roughly speaking, a p-value of 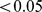 indicates that the linear correlation was significantly different from zero. Second, we calculated a linear fit (via standard linear regression) between the TDMI and the percentage change in the parameters, to assess how variation changed the TDMI — whether increasing/decreasing a given parameter increased/decreased or did not change the TDMI. The results are shown in Fig. 5 and detailed in table 1.

The results of this analysis are shown in Fig. 5 which details the effect the variation in parameters had on the TDMI distribution. After processing the sum of the parameter variation, the following conclusions can be drawn. TDMI increases with insulin independent glucose utilization (IIGU) (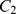, 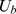), insulin secretion (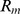, 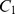, 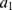), plasma insulin degradation rate (filtering rate by liver and kidneys) (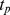), the delay between plasma insulin and glucose production (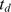), and glucose volume 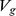. In contrast, the TDMI decreases with insulin dependent glucose utilization (IDGU) (in general) 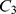, 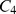, 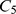, 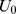, 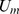 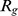 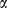, 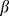. The faster insulin is filtered, the faster glucose is utilized independent of insulin, and longer the delay between plasma insulin and glucose production, the higher the predictability of glucose (this in turn implies faster glucose/insulin dynamical response). The more the system is dependent on insulin to cope with glucose, the slower the filtering of insulin, and the faster the reaction between plasma insulin and glucose production (e.g., by the liver), the less predictable the glucose. From a more dynamics perspective, the faster the glucose dynamics, meaning the quicker the oscillations between high and low levels of glucose coupled with a faster damping of the oscillations, the more predictable the glucose time series.

**Mechanistic explanation of the variation in the TDMI:** Broadly, there are three dynamical changes that are controlled by the parameters in the parameter ranges of 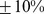: **(i)** a change in the *amplitude* of the finite-time steady state oscillation of glucose; **(ii)** a change in the *damping rate* on the finite-time oscillation, which decreases the amplitude of oscillation and more importantly, induces a *bifurcation* in finite-time glucose dynamics from a periodic orbit to a fixed point; and **(iii)** a change in the mean glucose value. To observe these dynamical changes in action, consider *five* test parameters, 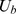, 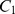, 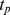, 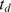, and 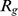 which control **(i)** IIGU, **(ii)**insulin secretion, **(iii)** kidney/liver filtration rates, **(iv)** delay between plasma insulin and glucose production, and **(v)** IDGU respectively.

**Figure 6:**
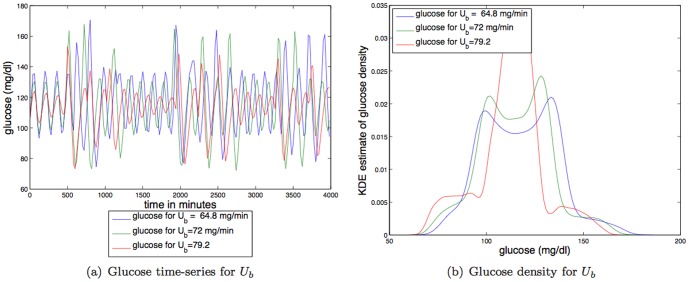
Depicted above are: (a) glucose time series for three different values of a linear constant affecting IIGU, 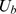; (b) glucose time series density for three different values of a linear constant affecting IIGU 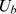.

**Figure 7:**
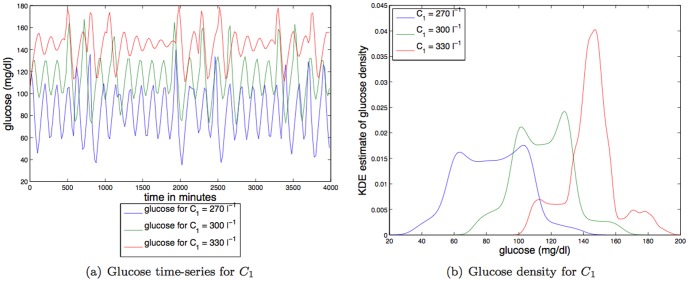
Depicted above are: (a) glucose time series for three different values of an exponential constant affecting insulin secretion, 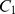; (b) glucose time series density for three different values of an exponential constant affecting insulin secretion, 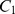.

**Figure 8:**
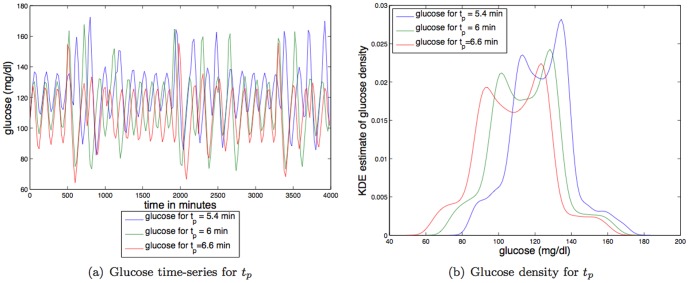
Depicted above are: (a) glucose time series for three different values of a time constant for plasma insulin degradation (via kidney and liver filtering), 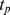; (b) glucose time series density for three different values of a time constant for plasma insulin degradation (via kidney and liver filtering), 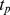.

**Figure 9:**
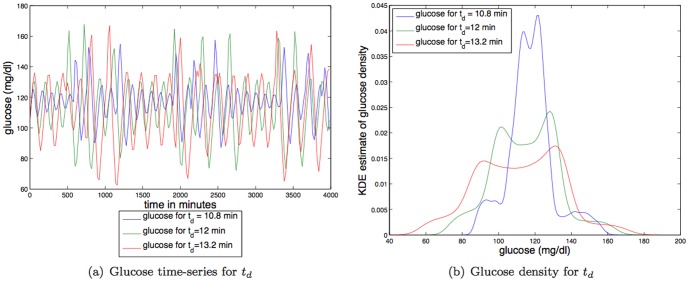
Depicted above are: (a) glucose time series for three different values of the delay rate between plasma insulin and glucose production, 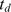; (b) glucose time series density for three different values of the delay rate between plasma insulin and glucose production, 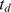.

**Figure 10:**
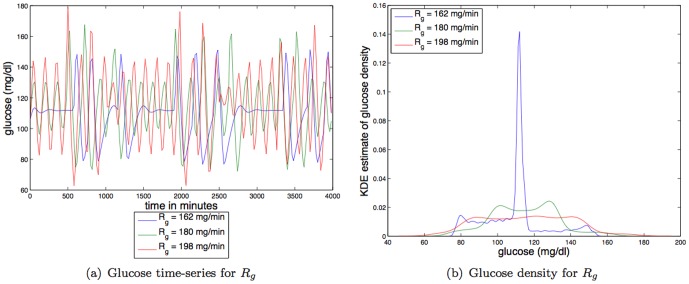
Depicted above are: (a) glucose time series for three different values of a linear constant affecting IDGU, 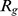; (b) glucose time series density for three different values of a linear constant affecting IDGU, 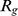.

- **Insulin independent glucose utilization (IIGU).** Figure 6 shows that increases in IIGU (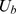) decreases the *amplitude* of the steady state oscillations, making the glucose distribution less like a uniform distribution, and thus increasing the TDMI (predictability). For parameter variation of 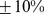 the TDMI varies about an order of magnitude (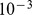 to 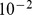); thus variation in IIGU has a reasonably strong effect on predictability.
- **Insulin secretion.** Figure 7 shows that increases in 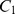, which *decreases insulin secretion*, changes two dynamical features of glucose. First, increases in 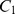 increase the mean glucose value which does not change the TDMI; changing the mean glucose level likely has a significant effect on the health of the individual. Second, increases in 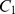 *increase the rate of damping* of the steady state oscillation, thus changing the short term dynamics of the system from an oscillation to a fixed point. Changing the damping rate, and thus the finite-time dynamics, *has a substantial* effect on the TDMI by making the glucose distribution more of a sharp, unimodal peak that, combined with the dynamics, induces a *higher* TDMI (predictability). For parameter variation of 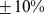 the TDMI varies about an order of magnitude (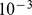 to 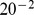); thus variation in insulin secretion has a reasonably strong effect on predictability.
- **Kidney and liver function and filtering rate.** Figure 8 shows that increases in 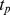, which *increases the filtration rate in the kidney/liver*, changes two dynamical features of glucose. Specifically, increases in 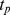 *decreases* the mean glucose value and *increases the amplitude* of the steady state oscillation. Thus, increases in 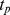 have approximately the opposite effect of increases in IIGU. Decreasing the mean glucose value does little to change the TDMI; it likely has a significant effect on the health of the individual. In contrast, increasing the amplitude of oscillations makes the glucose distribution more like a uniform distribution, which, combined with the dynamics, induces a lower TDMI. For parameter variation of 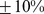 the TDMI varies by about a factor of 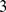 (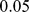 to 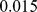); thus variation in kidney and liver function and filtering rate has a relatively weak effect on predictability.
- **Delay between plasma insulin and glucose production.** Figure 9 shows that increases in the delay between plasma insulin and glucose production (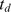) increase the *amplitude* of the steady state oscillations while slowing the glucose dynamics, making the glucose distribution more like a uniform distribution, and thus *decreasing* the TDMI. The effect of increasing the delay between plasma insulin and glucose production is the *opposite* from the effect of increasing IIGU. For parameter variation of 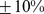 the TDMI varies about an order of magnitude (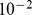 to 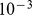); thus variation in the delay between plasma insulin and glucose production has a reasonably strong effect on predictability.
- **Insulin dependent glucose utilization (IDGU).** Figure 10 demonstrates how changes in the IDGU have a dramatic effect on the TDMI and on the glucose dynamics. The IDGU has several parameterizations, all of which are self consistent; however, to simplify the analysis, we will concentrate on 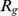, which affects the IDGU in a linear, and relatively simple way (via 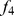). Decreasing 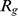 from the nominal value sharply increases the damping on the glucose and changes the finite time dynamics from a periodic orbit to a fixed point, thus greatly increasing the TDMI. Similarly, increasing 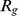 from the nominal value increases the amplitude of the steady state oscillation, decreasing the TDMI. It is possible that continued increase in the 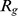 would eventually decrease the TDMI. For parameter variation of 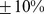 the TDMI varies about an order of magnitude times about 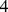 (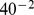 to 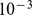); thus variation in IDGU has a relatively strong effect on predictability.

The surprisingly consistent results of the parameter variation analysis are shown in table 2. The mean glucose value does not have a strong effect on the TDMI more or less by definition *when the mean is constant for the entire patient record*. *The TDMI is elevated when the marginals are dependent and are not uniform distributions*. Because of this feature, changing the amplitude of the finite-time steady state, or the decay rate to a different finite time steady state (a fixed point), do have a substantial effect on the TDMI. However, it is the change in the amplitude of oscillation of the finite-time steady state oscillation that has the biggest effect on the TDMI. Because the strength of the oscillation is determined largely by the *delayed feedback control* within the endocrine system, the TDMI is a proxy for how well the endocrine system is maintaining the finite-state oscillation. Specifically, the TDMI seems to be minimized when the oscillation is strong (e.g., large amplitude, no decay to a fixed point). Put into more biological terms, the faster insulin is filtered, the faster glucose is utilized in a way that is dependent on insulin, and the faster the reaction between plasma insulin and glucose production (e.g., via the liver), the *lower* the predictability of glucose. The more insulin is secreted, the more glucose is removed independent of insulin, and the slower insulin is filtered by the kidneys and liver, the *higher* the predictability of glucose.

**Table 2:** Summary of the effects of various key parameters on the glucose dynamics, and TDMI that are observed when varying a parameter from 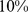 below the nominal value to 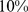 above the nominal value.

Glucose model parameters and their TDMI relationships					
Physical effect	parameter	amplitude of oscillation	decay rate	mean glucose	effect on TDMI
IIGU	*U_b_* 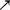	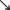	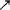	—	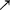
Insulin secretion	*C*_1_ 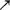	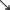	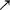	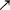	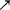
Kidney/Liver filtration	*t_p_* 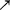	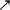	—	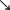	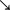
Delay between plasma insulin and glucose production	*t_d_* 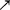	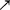	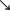	—	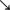
IDGU	*R_g_* 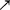	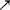	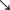	—	

The analysis above assumes that the model patients are stationary in the sense that their dynamic type does not vary because their parameters do not vary in time. In real situations captured in EHR data, this assumption is often violated. For instance, as is explained in Ref. [7], a single patient whose mean glucose has large variation *can* have a profound effect on the TDMI. Specifically, the TDMI can capture and represent the different mean glucose steady states (e.g., a mean of 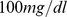 versus 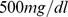) while missing many of the other, more subtle effects on the TDMI due to parameter variation. In this way changes or differences in both insulin secretion and liver and kidney function can dominate the estimated TDMI.

**Figure 11:**
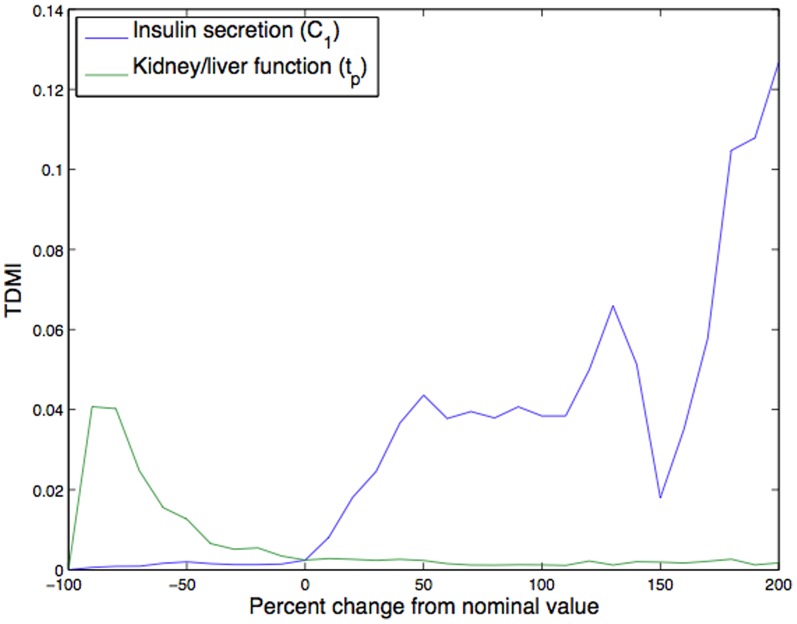
Depicted above are: the variations in TDMI for insulin secretion, 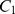, and kidney/liver function, 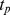, when varied by up to 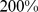 of their nominal values. Note that both undergo at least one bifurcation (qualitative state change) over this variation in parameters.

**Extended TDMI analysis of two clinically important parameters: kidney function and insulin secretion:** In a practical sense, mean levels of glucose are important. For instance, clinicians sometimes conceive of glucose as being in gross categories (low, normal, high, etc.) in accordance with the acuity of the patient. Thus far, none of the parameter variations we have induced changed the *mean* glucose level outside of normal ranges. What we did do is achieve an understanding of how variations in the 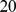 parameters affect the model glucose dynamics, glucose mean, and the TDMI. Nevertheless, to drive the model to have glucose values ranges that indicate differences in endocrine health that would appear in EHR data, we must alter the parameters that affect the mean glucose values more drastically. The two parameters that control mean glucose levels in the most acute way are kidney/liver function (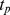) and insulin secretion (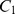). Focusing on these parameters, Fig. 11 depicts the TDMI variation when these two parameters are varied within 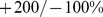 of their nominal values (note, neither parameter can be decreased by more than 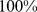 of its nominal value). Further note that insulin (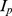) must remain positive; if we reduce 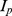 to a one dimensional ODE, qualitatively 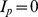 is either a attractor or a repellor.

**Figure 12:**
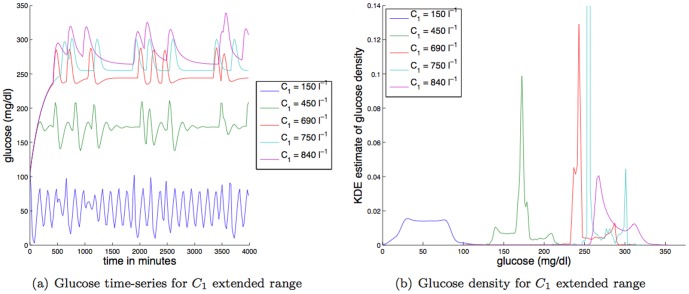
Depicted above are: (a) glucose time series for different values of the constant affecting insulin secretion, 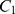; (b) glucose time series density for different values of the constant affecting insulin secretion, 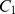.

**Large changes in insulin secretion:** As can be seen in Fig. 12, large changes in insulin secretion (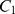) change both glucose dynamics and glucose levels. Specifically, even a 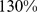 increase in 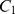 can drive glucose into an unhealthy range. Moreover, with the increase in mean glucose also comes drastic changes the glucose dynamics from a weakly damped oscillator that relaxes to an oscillatory state to a very highly damped oscillator that relaxes single value. Further increases in 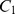 do generate bifurcations (cf. Fig. 11), but the overall dynamics and mean glucose effects change in a roughly monotonic way. Large decreases in 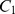 do not change the dynamics nor the mean glucose value in a dramatic way (note, there is a lower bound for 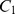). More mechanistically, focusing on the 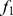 term that controls insulin secretion, increases in 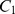 make 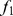 more negative per a fixed glucose (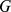) value, thus removing insulin from the blood more quickly, forcing both the glucose levels to rise and the distribution of glucose values to become more peaked (cf. Fig. 12). The effect increases the TDMI but not necessarily monotonically.

**Figure 13:**
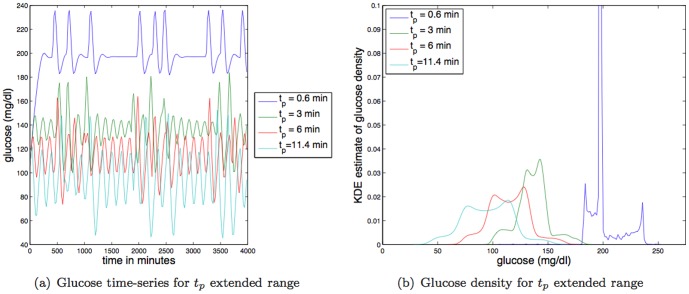
Depicted above are: (a) glucose time series for different values of the constant affecting kidney/liver function, 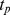; (b) glucose time series density for different values of the constant affecting kidney/liver function, 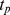.

**Large changes in kidney and liver function and filtering rate:** As observed in Fig. 13, decreases in kidney and liver function and filtering (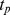) have a sharp effect on the dynamics and the glucose levels. For instance, an 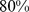 decrease in 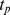 drives the mean glucose value up into the unhealthy range. Moreover, as was the case with increases in 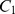, the increase in mean glucose accompanies drastic changes the glucose dynamics from a weakly damped oscillator that relaxes to a periodic-like orbit to a very highly damped oscillator that relaxes to a single value. In contrast, a 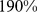 increase in 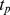 does little to change the glucose dynamics, although the glucose levels are driven down to some extent. Focusing more mechanistically on the term that governs how the kidneys and liver remove insulin from the blood, 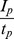, increases in 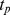 from zero increases the strength of the attraction of plasma insulin (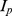) to zero. Therefore, increases in 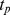 increases the rate at which insulin is removed from the bloodstream which forces both the glucose levels to rise and the distribution of glucose values to become more peaked (cf. Fig. 13). The effect of decreasing 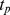 increases the TDMI monotonically.

*Summarizing*, decreases in insulin secretion (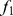 via *increases* in 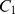) and decreases in the rate plasma insulin is filtered (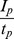 via *decreases* in 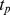) have similar effects as both decrease the amount of insulin present in the blood, increasing the amount of glucose in the blood, and destroying the oscillatory steady state of plasma glucose. This effect is confirmed by the effects on the TDMI shown in Fig. 11. Nevertheless, visual inspection of Figs. 12 and 13 shows that while there is a change in the mean, the glucose dynamics are affected slightly differently. Specifically, decreases in 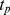 retains a part of the oscillation in glucose while increases in 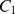 induces an immediate return to the fixed point steady state.

#### 0.9.3 NLP-based analysis of the 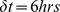 TDMI distribution

We extracted all the notes of the patients in our data set and experimented with their content similarity, roughly defined as the amount of content overlap between two records. The goal was to assess whether it is possible to separate the patients in the data set based on the content of the notes written in their longitudinal record. The records had approximately 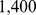 notes on average (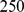 min, 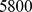 max, 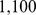 stdev) and spanned 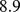 years on average (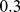 min, 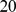 max, 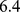 stdev).

To test whether patients can be separated based on their clinical characteristics, we extracted all mentions of disorders in their longitudinal records [18]. Disorders include names of conditions (e.g., hypertension) as well as signs, symptoms, and findings (e.g., edema, fever). Overall, the vocabulary of disorder concepts extracted from the notes in our dataset consisted of 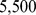 concepts. Longitudinal records had an average of 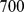 different disorders (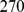 min, 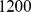 max, 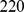 stdev).

When comparing the space of disorder mentions across all patients through a pairwise cosine similarity metric [19], records could not be separated according to their cosine similarity. For instance, consider two clusters, where one cluster contains the 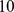 records with the highest TDMI, and the other contains the 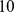 records with the lowest TDMI. We indexed the disorders in all the notes of the patients according to their 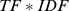 scores [19]. The 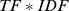 score for a given disorder 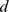 and a particular patient 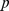 is a composite score which combines two weights: (i) the term frequency (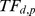) of the disorder in a particular patient (i.e., how many times 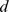 is mentioned in the patient record 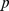); and (ii) the inverse document frequency (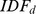) defined as 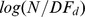, where 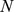 is the number of patients in the datasets, and 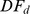 is the document frequency of 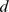, that is the number of patient records in the dataset which mention the disorder 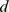. As such, the 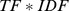 score for 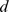 in patient 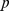 is low either if it is an infrequent disorder in the record or if many patient records in the dataset also mention the disorder. Conversely, the 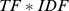 score will be high if a disorder is frequently mentioned within a record and the disorder is rarely mentioned in the records in the dataset.

We computed all pairwise similarities amongst the 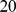 records. We found that the average intra-cluster similarities (the 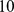 records in cluster 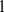 had on average a pairwise similarity of 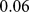, the 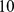 records in cluster 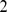 had on average a pairwise similarity of 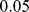) were the same as the average inter-cluster similarity (on average 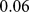), that is the similarity values did not correlate with TDMI. The same result was observed when indexing only the Signout and Progress notes in the records rather than all the notes (these note types are written by clinicians when a patient is in the hospital and summarize all the events during a particular shift), and when indexing based on all the words in the notes rather than disorder mentions.

The automated analysis of the patient narratives shows that basic, time-agnostic metrics do not allow to compare patients effectively, when trying to separate patients according to their stability in time.

#### 0.9.4 Manual-review-based analysis of the 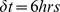 TDMI distribution

To attain a clinical interpretation of the TDMI clusters, we (GH and AP) performed a blind manual review of select patient records.

To begin, GH was given two lists of patient medical record numbers (MRNs) to review and cluster, data set *a* which had 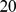 patients selected randomly from the four cluster grouping (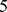 per cluster), and data set *b*, which had 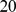 patients randomly selected from the two cluster grouping (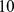 per cluster). The patients in each data set were randomly ordered, and GH was not shown the TDMI values. GH then reviewed the electronic medical records of each patient and grouped them according to factors likely to be correlated with glucose dynamics. This included the patient's age, type of diabetes (type 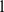 or type 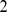), glucose levels, medications, severity of illness, feeding type (intravenous, enteral, normal, etc.), state of pancreas (e.g., failing, not failing, etc.), and other diseases (particularly those that affect glucose). GH grouped them by clinical similarity rather than attempting to rank them by suspected predictability. To assess the degree of agreement between the TDMI clusters and the expert-derived clusters, a p-value was obtained by generating random cluster assignments and estimating the distribution of the degree of overlap between the two clustering methods.

There was found to be no significant clustering for the four cluster grouping. GH then made the following expert-based categories: **C1** no explicit diabetes, no chronic insulin, may have some glucoses in the 200 and over range due to hospitalization, and may have some temporary insulin; **C2** diabetes of type 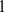, type 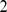, or steroid induced, with several glucoses over 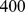, and many in the 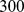
s, clearly repeatedly out of control; and **C3** diabetes (type 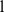, type 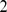, known steroid induced) well controlled with vast majority of glucoses under 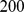. These three clinically defined categories were reduced to two categories of glucose dynamics, patients with episodes out-of-control glucose (C2) and patients with glucose in control (C1 and C3). *These clusterings of patients precisely predicted the TDMI clustering* (the categorization was 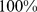 accurate).

The C1 and C3 patients all had low TDMI values, while the C2 patients had high TDMI values. We hypothesize that the intra-patient variability of the C2 patients makes the patients behave as a diverse population (patients with high and low glucose dynamics), driving up their TDMI values. In contrast, the C1 and C3 patients behave like homogeneous populations with similar glucose dynamics on the six-hour time windows. This would imply that to resolve more fine scale glucose dynamics would require filtering the patient population, restricting to only patients with in-control glucose values. The methodology and reasoning for this argument can be found in Albers and Hripcsak [7] (specifically, compare the results for data sets 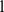 or 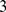 with data set 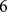).

To verify this categorization, we extracted another (independent) 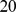 patient data set from the two cluster grouping and had AP categorize the patients according to C1, C2, and C3 without knowledge of the TDMI. AP's analysis matched GH's results with a single exception, one patient was classified as C1 who seemed to belong in the C2 category. Upon reviewing the glucose values of this patient, we found that the patient had a single glucose value that was deemed an outlier (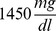). Such an outlier would cause the TDMI to be high for the same reason the TDMI was high for the C2 patients.

*Summarizing*, using a manual review we achieved a clinically relevant explanation for the TDMI clusters. Specifically, the high TDMI clusters correspond to patients who have episodes of out-of-control glucose and the low TDMI clusters correspond to patients with in-control glucose values regardless of disease (e.g., type 1 or 2 diabetes).

## Discussion

### 1.1 Key results

**Time matters** when studying human physiology or human health. While this fact seems obvious, time is rarely used in the study of health using EHR data. Here we demonstrate one instance where time can be important — we stratify patients by health using *derived measures* of physiologic variables that have time as a key parameter. Written differently, we can use dynamic information to derive a phenotype. Specific to this paper, predictability of glucose over a six hour time window for a random set of patients cleaved the population into a set of patients with episodes of in- and out-of-control glucose values (high TDMI), and patients with in-control glucose values (low-TDMI). Thus, the high TDMI indicates a diversity of health states, which serves as a proxy for higher acuity in the context of endocrine dynamics.

**Raw glucose values** do have meaning, especially to a clinician attending to a patient, but when integrating over an entire record and then an entire population, it seems that the derived values that incorporate time such as the TDMI or the LLC [20] are more useful for understanding and stratifying patients in a broad context.

**Mechanistic physiologic models** can be used to explain the physiologic sources of the variation in EHR data, here via the TDMI. Tying physiologic changes to disease *outcomes* is a more complex problem yet to be solved.

**The patient notes**, which greatly increase the breadth and power of EHR data, are a complex and difficult data set to leverage. In some sense, the notes can be considered a gold standard because they represent a written (often free-text, and thus quite expressive) representation of a patient at a time. On the other, the notes are *not a gold standard* because they are collected for clinical purposes and only include clinically relevant observations whose very relevance is biased by the clinician's training, opinions and time constraints [21]. Information about the patient is not captured in the notes in the same way that a scientist would record observations in a controlled setting. Moreover, temporality in the context of patient notes is a complex phenomenon: the narrative in a note can refer to multiple time points in the past and the future, some mentioned directly (e.g., “MI 09/02”, which conveys that the patient had a myocardial infarction in September 2002), some indirectly (e.g., “rash two days after surgery”), and some in an approximate fashion (e.g., “cat scan 3 weeks ago” means the patient had a cat scan approximately three weeks ago). Capturing these temporal expressions and resolving them on a patient timeline remains an open research topic for now [22]. Thus, in our analysis of the patient notes, while we leveraged the concepts to understand the clusters of TDMI values, we did not carry out a full temporal-aware analysis of the patient notes.

### 1.2 EHR-NLP based analysis in the context of physiology

Clinical decision support systems, augmented with knowledge extracted from the notes, have much promise to help clinicians with diagnosing a patient or making decisions on plan of care [23]. Thus, most of the research in NLP for clinical notes occurs in the context of information extraction (e.g., recognizing phrases in the text of patient notes denoting particular concepts, like a disorder, medication, or laboratory test). NLP of clinical text is challenging because of the underlying linguistic characteristics of clinical language (see [24] for an overview of NLP in the medical domain). There is much lexical ambiguity in clinical texts; for instance, the string “2/2” can refer to a date (February 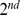) or the abbreviation for “secondary to,” “HF” can mean “heart failure” or “hispanic female.” Furthermore, because of the presence of free text, typos and mispellings can be found in a note, complicating the extraction process when relying on existing dictionaries of medical terms like the UMLS [25] as gold standard. More critically, from a semantics standpoint, because free text has a large power of expressiveness, there are many ways to refer to the same piece of information, or concept. For instance, the presence of type 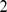 diabetes in our data set of notes was conveyed in a varied number of ways, including “DM2,” “diabetes,” “t2dm,” and “type II diabetes,” as well as less direct phrases such as “blood sugar” and “hyperglycemia,” or even names of diabetes medication like “glucophage.” Not all variants are included in existing medical dictionaries (of the ones mentioned for diabetes for instance, only three variants are listed in standard dictionaries). Finally, the mention of a concept alone is not enough to determine whether the concept is actually relevant to a patient under examination – other linguistic indicators, or modifiers, affect the meaning of the concept. For instance, presence of negation (itself conveyed through many alternative phrases such as “absence of” or “patient denies” or ambiguous abbreviations like “-”), uncertainty (e.g., “possible” or “suggests”), and temporal expressions (“history of” vs. “current” vs. “risk of”) are important to recognize and process in the note to get an accurate semantic representation of a note. We are not aware of work linking NLP of clinical notes to physiology or concepts dealing with physiology in a patient note.

While the results presented in this paper indicate that the NLP analysis could not separate the patients in the same way as the TDMI analysis, future work will consider the impact of incorporating the temporal signals of disorders within the TDMI analysis of glucose, and investigate whether this refines the clusters even further.

### 1.3 TDMI-based interpretation of the clinical and physiologic results

#### 1.3.1 Small variations in the TDMI and glucose dynamics and its clinical interpretation

Focusing on Fig. 5 which details the effects of 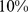 in parameter variation on the TDMI, we have several *potentially* clinically relevant explanations related to the TDMI variation. The less one's body requires insulin to process glucose, say, through exercise, higher metabolism, or lower insulin resistance, the faster one's glucose/insulin dynamics, and the more predictable one's glucose/insulin dynamics become over time scales shorter than 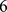 hours on a *fine scale* (i.e., neglecting large intra-patient changes in health state). *More fundamentally*, this phenomena is not limited to type 2 diabetes; we know this because the manual review, which did take type 2 diabetes into account, was not able to separate the population due to type 2 diabetes (meaning, the population didn't separate into patients with and without type 2 diabetes). Moreover, from considering Figs. 6–10 it is clear that the TDMI can change for a multitude of reasons that change the distribution of glucose values. There are two notable reasons why this is important. *First*, there is a diversity among the patients within this particular data set, and therefore among these patients there are many reasons why a patient can be highly dependent on insulin to process glucose, or have impaired filtering mechanisms (e.g., impaired liver and kidneys). This means *the mapping between physiologic dynamics and disease is complex, many-to-one, and not necessarily onto*. For instance, one patient with type 2 diabetes that is managed well can have a similar health state as a patient without type 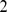 diabetes, while a different patient with type 2 diabetes can have a health state similar to a patient with a severely failing pancreas without type 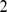 diabetes. More generally, a broad phenotype (e.g., type 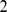 diabetes) whose specification is binary cannot quantify severity of acuity, while measures such as glucose predictability can. *Second*, mechanistic models do not explicitly have health states built in, but rather have physiologic processes that affect glucose and insulin dynamics — the health state is a result of the functioning of these processes and possibly many others. Here it just so happens that poor health states (poor filtering, insulin resistance, high level of insulin required to process glucose) correlates with the same characteristics that lead to low predictability of glucose as quantified by the TDMI. The situation could have easily been reversed, or even shown no correlation at all. *Finally*, these conclusions do provide a compelling match to type 2 diabetes and connect this constructive model to longer term physiologic/pathophysiologic dynamics — *this is a surprising result given that the model was designed to work over time periods of minutes to hours*.

The stark conclusion then is that EHR laboratory data are capturing glucose dynamics and health at a finer scale than medical professionals are recording in the notes using broad phenotypes. Specifically, degree of acuity often goes unmentioned in the patient notes, but EHR glucose data does seem to synchronize with physiology/pathophysiologic models in such a way that one can *stratify patients into different health states using TDMI-specified predictability of glucose*. It goes without saying that there is much left to understand regarding these results; moreover, this analysis highlights to why it is critical to understand the population and to model the collection and representation of EHR data. These results give hope that through the combination of modeling and EHR-data analysis, better treatment though a combination of EHR-data-driven analysis and physiologic modeling is possible.

#### 1.3.2 Large variations in the TDMI and glucose dynamics and its clinical interpretation

The primary clinical observable related to glucose is its magnitude; in a clinical setting glucose dynamics are not normally assessed and are difficult to measure on a time scale faster than an hour (in the near future we will be able to measure glucose continuously in a clinical setting). There were three model parameters that affected the magnitude of the glucose, caloric intake (exogenous glucose delivery), insulin secretion (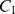) and kidney/liver function (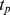). The dominant TDMI feature that is observed in EHR data and is relatable to the physiologic model is known to be driven by nutrition [6]. Stratifying the population by the TDMI signal beyond nutrition, we found the variation in the TDMI could be driven by health state. The form of the health state was, however, surprising. Instead of the TDMI stratifying the population by disease, the population was stratified into two modes corresponding to populations with both in- and out-of-control glucose dynamics, and populations with in-control glucose dynamics. According to the mechanistic model, glucose can be driven out of control primarily through insulin secretion and kidney/liver filtration. In both cases, removing (or rendering ineffective) insulin too quickly causes a rise in glucose. Moreover, increasing insulin secretion (increasing 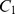) and increasing the filtration of insulin (decreasing 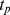) had identical effects on the TDMI and mean glucose values. Therefore, it is likely that implicitly, it is the time varying insulin secretion, which is parameterized by three parameters, that led to the out-of-control, nonstationary nature of the dynamics of the high-TDMI cohort. The question remains as to whether we can do more fine scale physiologic analysis with EHR data, either by refining the population *a la* directed dynamical phenotyping or by generalizing to a multi-variate situation in the undirected phenotyping context. In either case, it seems that EHR data will be helpful in model refinement, including generalizing the model we use here into more pathophysiologic contexts.

### 1.4 Future exploratory analysis: connecting physiology and clinical practice

This paper is primarily about using EHR data in the context of mechanistic physiologic modeling. Specifically, we want to use EHR data to refine, develop, and use mechanistic modeling. The results here suggest broad problems that we can now begin to solve.

**Tying dynamics to outcomes:** It is likely that the single greatest key to connecting physiology to clinical practice in the way that physics has been unified with engineering is to tie physiology to macroscopic outcomes. Inherently, this implies using temporal analysis to cleave and understand different groups of patients because health outcomes evolve implicitly over time. There are numerous ways of achieving this goal. As an example, consider the question: do fast time (order minutes) glucose dynamics matter for long term health? As can be seen in Fig. 10, changes in IDGU though 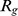 can have a profound impact on the observed glucose dynamics. Specifically, decreases in 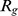 tend to decrease glucose oscillation whereas increases in 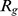 tend to do the opposite. The question then becomes, do these oscillations in glucose have an impact on the long term outcomes such as the health of the individual? Here the dynamics are probably indications if not causes of poor outcomes, but such an assertion remains to be shown with data. These types of questions may play a significant roll in discovering, for instance, the optimal means of administering nutrition to individuals in the ICU that lead to the optimal outcomes.

**Forecasting future health using data assimilation:** Data assimilation [26] [27] combines *observed* data from the current (and often the past) state(s) of the system with underlying dynamical principles governing the system (i.e., a constructive model) to make an accurate estimate or forecast of the true state of the system at any given time, *including variables that were not measured*. From a more practical standpoint, DA schemes perform two functions: **(i)** they reconstruct the state variables of a model, including both observed and unobserved variables; and **(ii)**, they forecast the future in a way that can be directly tested with future measurements (and used to implement control theory). This allows for “patient forecasts,” where different outcomes can be based on current and future observations and/or hypothetical data, allowing for exploration of “what if” scenarios with patients. This in turn allows us to take a more personalized view of treatments for patients in clinical applications. Finally, some DA schemes (e.g., unscented Kalman filters) allow for “empirical observability,” or the ability to *rank* which variables are the most useful for reconstructing the other variables, allowing us to determine the most useful clinical variables, in some sense. Sedigh-Sarvestan *et al* [28] applies a DA applied to the model in this paper that includes empirical observability ranking of parameters and variables.

**Designing optimal treatment using control theory:** Control theory [27] [29] [30] [31] applied to solve biomedical and clinical problems has a very successful but limited history. Examples include implantable cardioverter-defibrillator or pacemakers to cope with irregular heartbeats, work toward creating an artificial pancreas [32], and to design treatments for prostate cancer [33]. To apply (optimal) control theory to any problem, one usually requires three components, an explicit model of the process to be controlled (e.g., the glucose/insulin model shown here), a statement regarding the constraints of the system (e.g., fixed or disallowed parameter settings, initial conditions, boundary conditions, etc.), and specification of the performance (e.g., how tightly one wants to control glucose) [29]. EHR data will likely be the only data available on a population scale that can be used to test a models, specify the constraints, and specify the desired performance (based on retrospective EHR-data based study) based on desired outcomes. With a control theory infrastructure in place for a given physiologic system applications are very broad. For instance, one could design a controller to regulate glucose in an ICU setting (cf. Sedigh-Sarvestan *et al* [28] where an unscented Kalman filter is applied to the model in this paper), one could use the controller to design optimal treatment strategies over long periods of time for outpatient type 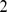 diabetics, or one could design artificial organs such as the artificial pancreas project [34] [35] [36]. But these possibilities are only possible in practice when we have a constructive model available as well as defined target dynamics that are tied with outcomes.

**In silico experimentation:** If a mechanistic model can be verified sufficiently well it can be used to test new drugs and treatments even without data (e.g., outside of a personalized medicine approach where data assimilation is used). Such a situation is referred to as *in silico* experimentation, and it has already begun in some contexts. For example, recently an endocrine model of the type 1 diabetes, being used in the context of developing an artificial pancreas [32], has been approved by the FDA as a substitute for animal trials for preclinical trials [36] [34] [35]. In this case, artificial data is created (based on real data, but not a DA analysis), and then different treatment strategies are tested. This approach has the potential to greatly accelerate the rate of advancement of therapy in many different contexts.

**Limits of EHR data:** EHR data are diverse and collected in an uncontrolled environment. What is the limiting dynamical resolution that can be observed through refining the EHR population, and what are the right ways to go about refining or filtering the population as is required for constructive dynamical phenotyping (cf. Fig. 1)? For instance, if we select patients whom are measured frequently, these patients will likely have high acuity; sometimes such measurement characteristics can be used to help identify phenotypes [17]. In this circumstance we many want to exclude certain diseases and include others. However, such filtering can limit and bias observations. The original hypothesis we formulated here was that we could split the population via a disease such as having diabetes (type 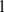 or 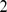) or not. This turned out to not be the primary force driving the difference in TDMI *in a broad EHR population*. Thus, the filtering of the population and the discovery of the limits of what EHR data can verify are non-trivial problems.

**Multivariate dynamical phenotyping:** In this paper we used a single signal to stratify the population and test the model. Disease state is almost never univariate; complex phenotypes are always multi-variate and multi-scale. Thus, it is likely that dynamical phenotyping, especially in the case of undirected dynamical phenotyping (cf. Fig. 1), will require a multi-variate approach, leading to the question, what will constitute the most useful variables for stratifying and understanding a population given the constraints of EHR data. Currently, very few temporal processing techniques have been adapted for the EHR data context [37]; some traditional time-to-event techniques are being developed to apply to EHR data [38].

**Deconvolution of complex biases from EHR and data:** To forge a more practical relationship between physiology and applying physiologic principles in a clinical setting, we must tie physiologic dynamics to observable outcomes. To associate physiologic dynamics to observable outcomes, we must have a diverse and large enough population to allow for the calculation of convergent, meaningful statistical quantities. EHR data may be the only data set that will be large and diverse enough to discover physiologic connections to outcomes in concrete ways because of its uncontrolled and broad nature. Nevertheless, with uncontrolled nature of EHR data also comes complex biases [2]. For instance, here we stratify the population not by disease, but by the *intra-patient* diversity of glucose dynamics. This stratification is, in some sense, an EHR bias because the stratification is based on the fact that a single patient can simultaneously represent multiple health states. Because of this, as well as other reasons such as the fact that many diseases can lead to the same physiologic effect in a subset of the human systems (e.g., the endocrine system), the *stratification by dynamics* or dynamical phenotyping using *a single variable*, does not cleanly map according to clinical notions of disease. This is the reason why we suggest moving to multi-variate dynamical phenotyping as a possible solution in the context of *undirected dynamical phenotyping*.
